# Use of Allied Health Services in Rural Northern Victoria, Australia

**DOI:** 10.1111/ajr.70001

**Published:** 2025-02-03

**Authors:** Andrew J. Hamilton, Ryan McGrath, Lisa Bourke, Kristen M. Glenister, David Simmons

**Affiliations:** ^1^ Department of Rural Health, Melbourne Medical School University of Melbourne Shepparton Victoria Australia; ^2^ Allied Health Education and Research Unit Goulburn Valley Health Shepparton Victoria Australia; ^3^ School of Allied Health, Exercise and Sports Sciences Charles Sturt University Thurgoona New South Wales Australia; ^4^ Department of Rural Health University of Melbourne Wangaratta Victoria Australia; ^5^ Macarthur Clinical School Western Sydney University Penrith New South Wales Australia; ^6^ Macarthur Diabetes, Endocrinology and Metabolism Service Campbelltown Hospital Campbelltown New South Wales Australia

**Keywords:** allied health occupations, audiology, optometry, physical therapy modalities, psychology, social work

## Abstract

**Objective:**

Little is known about the socio‐demographic factors associated with the use of allied health services in rural Australia. The objective of this study was to determine which factors were associated with the use of various modes of allied health in a region of Northern Victoria.

**Methods:**

This is a secondary analysis of the Crossroads‐II population health study. Generalised linear mixed models were constructed.

**Design:**

Households were selected at random through address local government area lists. Data were collected by door‐to‐door surveying.

**Settings:**

The northern part of the Goulburn Valley, Victoria, including one large rural conurbation (MM 3) and three medium rural towns (MM 4).

**Participants:**

Over 15 years of age.

**Main Outcome Measures:**

Use of allied health services.

**Results:**

The odds of using audiology (1.047 [1.035, 1.059]), optometry (1.034 [1.027, 1.042]) and podiatry (1.052 [1.039, 1.066]) increased with age, and psychology decreased (0.985 [0.974, 0.997]). Females had lower odds than males for audiology (0.708 [0.553, 0.907]) and greater odds for optometry (1.712 [1.421, 2.064]) and pharmacy advice (1.593 [1.317, 1.927]). Greater odds were observed forbeing Australian‐born and pharmacy advice (1.581 [1.149, 2.175]), English spoken at home and physiotherapy (2.415 [1.279, 4.560]), a bachelor's degree and psychology (1.579 [1.011, 2.466]) and pharmacy advice (1.296 [1.002, 1.675]), not working and psychology (3.518 [1.999, 6.191]) and social work (4.202 [2.110, 8.367]). Those unable to work had greater odds of using six of the eight services investigated.

**Conclusion:**

Socio‐demographic associations with allied health use vary across disciplines. For this population in rural Victoria, socio‐demographic associations were observed for all of the allied health modalities studied. Such relationships need to be studied in other rural and allied health contexts.

**Summary**

- What this paper adds
  ○ Relative to medical care, allied health services are little used in this part of regional Northern Victoria.
  ○ The relationships between various socio‐demographic factors and the use of allied health services in a region of Northern Victoria were studied.
  ○ These vary markedly across service types but are broadly similar for those of similar purposes and functions, such as physiotherapy and exercise physiology.
- What is already known on this subject
  ○ Allied health receives less advocacy and attention than nursing and medicine.
  ○ Factors associated with the use of allied health have received some attention in Australian urban settings but not in rural areas.

## 1 Introduction

Along with nursing and medicine, allied health is one of the three pillars of the patient‐care network in Australia [1]. It has been argued that it is the most overlooked of these, despite accounting for about one‐fifth of the health‐care workforce [1]. Overlaying this is an imbalance in the provision of services across regions of different rurality. In Victoria, for example, regional centres, large rural towns and medium rural towns have more podiatrists per 10 000 population than metropolitan regions, and regional centres and large regional towns have more occupational therapists by population than metropolitan areas [2]. In contrast, there are more physiotherapists per capita in metropolitan areas than in any of the regional zones [2]. Variation within regional areas can be significant, with larger towns not necessarily being better serviced. Nationally, the full‐time‐equivalent provision of physiotherapy, psychology, occupational therapy and optometry services is 14%, 29%, 28% and 5% greater in medium rural towns than in large rural towns [3].

In Australia, allied health is a rapidly growing sector, with high compound annual growth rates (2016–2021) of workforce, including pharmacy (2.5%), optometry (4.1%), podiatry (4.1%), psychology (4.5%), physiotherapy (5.6%) and occupational therapy (7.2%) [3]. Such statistics paint a broad picture of allied health use [3]. A fuller understanding can be achieved by disaggregating populations into socio‐demographic characteristics. Most Australian research on factors associated with the use of allied health has been in an urban or national context [4, 5, 6, 7, 8]. Although some studies of rural users have been undertaken, they are relatively few [9, 10]. These growing services have received more attention in the context of workforce and training than they have in terms of the people who use them [11, 12, 13, 14, 15].

Socio‐demographic factors associated with the use of allied health services can often be considered only individually, for large datasets on several factors are uncommon. The Crossroads‐II dataset offers the opportunity to consider several factors simultaneously and thus allows consideration of interactions among factors. Further, a multi‐factor understanding of allied health use is even rarer in rural settings. The other significant opportunity here is that different allied health services can be considered alongside one another.

The purpose of this study was to assess the importance of various socio‐demographic factors in terms of the use of several allied health services in a region of rural Northern Victoria, Australia. This was undertaken through analysing data from Crossroads‐II, which was a large population health survey in the region.

## 2 Methods

### 2.1 Study Design

This is a secondary analysis of data that were collected as part of the Crossroads‐II project. The design of Crossroads‐II is given in detail in a dedicated publication [16]. In short, door‐to‐door surveys were conducted in four towns in the Goulburn Valley of Northern Victoria, Australia, from October 2016 through to November 2018. Council residential rate‐payer lists were used for the random selection of 3657 households, which reduced to 3112 following the exclusion of unoccupied properties. Of these households, 1895 agreed to partake in the survey. This equated to 2679 persons: 473 from Benalla, 431 from Cobram, 431 from Seymour and 1344 from Shepparton Mooroopna. Only participants who had lived in or near the town for 6 months or more were interviewed. The minimum age of interviewees was 16 years. The demographics of the sample are given in Table 1.

**TABLE 1:** Demographic parameters of the sample population.

Age	Mean: 53.7 (range: 16–97)
Sex	Female: 461 (61%) Male: 294 (39%)
Highest level of education	Not finished Year 12: 1073 (42.1%) Year 12: 937 (36.7%) University: 540 (21.2%)
Primary language at home	English: 2354 (93.4%) Other: 165 (6.6%)
Town	Benalla: 473 (17.7%) Cobram: 431 (16.1%) Seymour: 431 (16.1%) Shepparton‐Mooroopna: 1344 (50.2%)
Employment	Full‐time: 729 (29.0%) Part‐time: 496 (19.8%) Not working (but not retired): 112 (4.5%) Student: 93 (3.7%) Retired: 810 (32.3) Unable to work: 101 (4.0) Other: 169 (6.7)

The relevant survey question was ‘In the past 12 months have you visited any of the health services listed below?’ The options included various health services, and of those, the following were identified as allied health: audiology, exercise physiology, optometry, pharmacy, physiotherapy, podiatry, psychology, social work, and speech pathology. Each of these is considered to be allied health by the Allied Health Professions Australia alliance [17].

### 2.2 Analysis

Frequencies of the use of the various allied health services were calculated and placed against general practitioner (GP), specialist and hospital outpatient services for comparative purposes.

Generalised linear mixed models (GLMM) were constructed to assess factors associated with the use of the various allied health services. *Town* was modelled as a random effect. The fixed‐effects model comprised the independent variables listed in Tables 2, 3, 4, 5. Given the dependent variables were Bernoulli distributed, the logit link function was used, and the fixed‐effect model is thus a binary logistic regression. This model included an intercept term, but the random‐effect model did not.

**TABLE 2:** Relationships of the use of audiology and optometry with the fixed effects in the GLMM.

Variable	Audiology (*n* = 2365)		Optometry (*n* = 2366)	
	*b*	OR [CI_95_]	*b*	OR [CI_95_]
Age	0.046	1.047 [1.035, 1.059]	0.034	1.034 [1.027, 1.042]
Sex (male)	−0.346	0.708 [0.553, 0.907]	0.538	1.712 [1.421, 2.064]
Born in Australia (no)	0.084	1.087 [0.735, 1.609]	0.285	1.330 [0.99, 1.788]
Private insurance (no)	0.285	1.330 [1.030, 1.718]	0.523	1.686 [1.396, 2.037]
English at home (no)	−0.248	0.780 [0.395, 1.543]	−0.332	0.717 [0.462, 1.113]
Education (no Year 12)	—	—	—	—
Year 12	−0.070	0.933 [0.695, 1.252]	0.175	1.191 [0.963, 1.474]
Bachelor's degree	−0.083	0.920 [0.651, 1.301]	0.099	1.104 [0.857, 1.421]
Employment (FT)	—	—	—	—
Part‐time	−0.262	0.770 [0.504, 1.176]	−0.022	0.978 [0.754, 1.270]
Not working	−0.633	0.531 [0.233, 1.213]	0.046	1.047 [0.670, 1.636]
Student	−0.302	0.739 [0.219, 2.502]	0.387	1.473 [0.866, 2.505]
Retired	−0.077	0.926 [0.612, 1.401]	0.368	1.445 [1.066, 1.958]
Unable	−0.497	0.609 [0.278, 1.333]	−0.219	0.803 [0.501, 1.288]
Other	−0.241	0.786 [0.403, 1.533]	−0.032	0.968 [0.659, 1.423]
Intercept	−4.123	0.016 [0.007, 0.038]	−2.670	0.069 [0.040, 0.119]

*Note:* Reference levels are presented in parentheses. The employment level ‘unable’ means permanently unable to work/ill.

Abbreviations: [CI_95_], 95% confidence interval for OR; *b*, rate of change in log‐odds; OR, adjusted odds ratio.

The Variance Inflation Factor (VIF) and variance decomposition were used to detect potential collinearity among independent variables. VIF values were determined subsequent to running a series of auxiliary linear regressions [18]. If the VIF for a given independent variable exceeded 10, then collinearity was deemed problematic in the fixed‐effects model [19, 20]. After decomposing the variances, condition indices (*η*) were calculated from the eigenvalues for each singular value. Collinearity was considered to exist if, for any singular value, *η* > 15 and at least two independent variables had variance proportions > 0.5 [21, 22].

Area under the receiver operating characteristic curves, classification accuracy, sensitivity, and specificity were used to assess the performance and utility of the models. Classification cut‐off values were determined through the Index of Union (IU) approach, which has the advantage of simultaneously maximising sensitivity and specificity [23].

SPSS (Version 29) was used for constructing the GLMMs. Sensitivity and specificity calculations accommodated the full models, not just the fixed‐effects models, and were calculated by hand, as were the IU values.

## 3 Results

The frequencies of use of the various allied health services were: audiology (14.5%), exercise physiology (2.8%), optometry (46.2%), pharmacy (32.5%), physiotherapy (18.2%), podiatry (12.9%), psychology (6.7%) and social work (3.6%). In contrast, medical care services were used more frequently: GPs (92.5%), specialists (43.2%), and hospital outpatient (20.2%).

There was a significant improvement in the null model for all modalities (*p* < 0.01) save speech therapy, which failed to converge and was therefore excluded. The odds of using audiology and optometry increased with age (OR 1.047 [1.035, 1.059]), which yields 1.6‐ and 1.4‐fold increases, respectively, for a 10‐year increase in age, with a 30‐year increase resulting in respective ORs of 4.0 and 2.7 (Table 2). Relative to males, females had greater odds of using optometry services and lesser odds of using audiology (OR 0.708 [0.553, 0.907]). Private health insurance increased the odds of using audiology (OR 1.330 [1.030, 1.718]) and optometry services (OR 1.686 [1.396, 2.037]). Retired people had greater odds of using optometry, but no employment effect was observed for audiology (*p* = 0.529).

The odds of home English speakers using physiotherapy were greater than for those without English as their primary language at home (OR 2.415 [1.279, 4.560]) (Table 3). Those permanently unable to work or ill had greater odds, relative to full‐time workers, of using physiotherapy and exercise physiology (OR 2.415 [1.279, 4.560]), and the odds of using physiotherapy were lower for those who were not home English speakers (OR 2.543 [1.565, 4.131]) (Table 3).

**TABLE 3:** Relationships of the use of physiotherapy and exercise physiology with the fixed effects in the GLMM. Presentation as per Table 2.

Variable	Physiotherapy (*n* = 2367)		Exercise physiology (*n* = 2365)	
	*b*	OR [CI_95_]	*b*	OR [CI_95_]
Age	0.004	1.004 [0.995, 1.013]	0.007	1.007 [0.986, 1.029]
Sex (male)	−0.074	0.929 [0.744, 1.160]	−0.113	0.893 [0.523, 1.526]
Born in Australia (no)	−0.104	0.557 [−0.451, 0.243]	−0.325	0.703 [0.331, 1.495]
Private insurance (no)	0.218	1.243 [0.989, 1.563]	−0.074	0.929 [0.515, 1.674]
English at home (no)	0.882	2.415 [1.279, 4.560]	0.965	2.624 [0.553, 12.461]
Education (no Year 12)	—	—	—	—
Year 12	0.182	1.199 [0.927, 1.551]	−0.267	0.766 [0.406, 1.445]
Bachelor's degree	0.297	1.345 [0.997, 1.816]	0.126	1.134 [0.550, 2.337]
Employment (FT)	—	—	—	—
Part‐time	0.020	1.020 [0.741, 1.404]	−0.947	0.388 [0.126, 1.198]
Not working	−0.040	0.960 [0.547, 1.686]	0.482	1.619 [0.510, 5.133]
Student	−0.124	0.883 [0.441, 1.768]	−10.486	0.181 [0.011, 2.938]
Retired	0.042	1.043 [0.721, 1.508]	−0.084	0.919 [0.369, 2.294]
Unable	0.933	2.543 [1.565, 4.131]	1.585	4.881 [3.030, 11.738]
Other	−0.277	0.758 [0.449, 1.277]	0.860	2.363 [0.929, 6.010]
Intercept	−2.678	0.069 [0.033, 0.143]	−4.528	0.011

The use of psychology decreased with age (OR 0.985 [0.974, 0.997]) (Table 4). This equates to a 12.5% reduction in odds for a 10‐year increase in age and a 26.1% reduction over 20 years. Those holding a bachelor's qualification had greater odds of using psychological services than those who had not completed Year 12 (1.579 [1.011, 2.466]). For psychology and social work, the odds were markedly greater for those not working (OR 3.518 [1.999, 6.191] and OR 4.202 [2.110, 8.367]) and those unable to work (OR 3.509 [1.910, 6.447] and OR 2.808 [1.247, 6.326], respectively) (Table 4).

**TABLE 4:** Relationships of use of psychology and social work with the fixed effects in the GLMM. Presentation as per Table 2.

Variable	Psychology (*n* = 2367)		Social work (*n* = 2367)	
	*b*	OR [CI_95_]	*b*	OR [CI_95_]
Age	−0.015	0.985 [0.974, 0.997]	−0.012	0.988 [0.974, 1.003]
Sex (male)	0.152	1.164 [0.836, 1.621]	−0.204	0.816 [0.554, 1.201]
Born in Australia (no)	0.432	1.541 [0.840, 2.825]	−0.179	0.836 [0.464, 1.506]
Private insurance (no)	−0.124	0.883 [0.626, 1.245]	−0.202	0.817 [0.539, 1.240]
English at home (no)	0.770	2.160 [0.832, 5.608]	0.466	1.594 [0.630, 4.037]
Education (no Year 12)	—	—	—	—
Year 12	0.260	1.297 [0.894, 1.884]	−0.056	0.945 [0.616, 1.451]
Bachelor's degree	0.457	1.579 [1.011, 2.466]	−0.132	0.876 [0.501, 1.533]
Employment (FT)	—	—	—	—
Part‐time	0.080	1.083 [0.691, 1.697]	0.306	1.357 [0.743, 2.480]
Not working	1.258	3.518 [1.999, 6.191]	1.436	4.202 [2.110, 8.367]
Student	0.050	1.051 [0.452, 2.448]	0.097	1.102 [0.367, 3.304]
Retired	−0.132	0.876 [0.485, 1.582]	0.528	1.696 [0.846, 3.399]
Unable	1.255	3.509 [1.910, 6.447]	1.032	2.808 [1.247, 6.326]
Other	0.137	1.147 [0.604, 2.181]	0.824	2.279 [1.105, 4.704]
Intercept	−3.248	0.039 [0.014, 0.108]	−2.797	0.061 [0.021, 0.181]

Those unable to work also had greater odds of using podiatry and pharmacy advice. Use of podiatric services was also characterised by a strong age effect (OR 1.052 [1.039, 1.066]) (Table 5). This equates to a 2.8‐fold increase for a 20‐year age difference and a 7.6‐fold increase for a 40‐year difference. In addition, those unable to work (OR 2.588 [1.653, 4.050]), females (OR 1.593 [1.317, 1.927]), people born in Australia (OR 1.581 [1.149, 2.175]), and those with a bachelor's degree (OR 1.296 [1.002, 1.675]) had greater relative odds of seeking pharmacy advice.

**TABLE 5:** Relationships of the use of podiatry and pharmacy advice with the fixed effects in the GLMM. Presentation as per Table 2.

Variable	Podiatry		Pharmacy	
	*b*	OR [CI_95_]	*b*	OR [CI_95_]
Age	0.051	1.052 [1.039, 1.066]	−0.005	0.995 [0.987, 1.002]
Sex (male)	0.207	1.230 [0.939, 1.609]	0.466	1.593 [1.317, 1.927]
Born in Australia (no)	0.063	1.065 [0.703, 1.611]	0.458	1.581 [1.149, 2.175]
Private insurance (no)	−0.038	0.963 [0.729, 1.272]	−0.042	0.959 [0.790, 1.165]
English at home (no)	0.838	2.311 [0.794, 6.726]	0.246	1.278 [0.791, 2.006]
Education (no Year 12)	—	—	—	—
Year 12	0.200	1.221 [0.892, 1.672]	0.142	1.153 [0.931, 1.428]
Bachelor	0.346	1.414 [0.974, 2.051]	0.259	1.296 [1.002, 1.675]
Employment (FT)	—	—	—	—
Part‐time	0.163	1.177 [0.716, 1.933]	0.098	1.103 [0.845, 1.440]
Not working	0.320	1.377 [0.628, 3.019]	0.340	1.405 [0.906, 2.180]
Student	0.067	1.070 [0.241, 4.745]	−0.547	0.578 [0.322, 1.039]
Retired	0.468	1.597 [0.985, 2.591]	0.270	1.310 [0.954, 1.797]
Unable	0.775	2.170 [1.097, 4.293]	0.951	2.588 [1.653, 4.050]
Other	0.096	1.100 [0.517, 2.340]	0.831	2.295 [1.589, 3.316]
Intercept	−6.372	0.002 [0.000, 0.006]	−1.581	0.206 [0.114, 0.370]

Random (town) effects were not observed for any of the models. They were insignificant for audiology (*p* = 0.724), exercise physiology (*p* = 0.332), optometry (*p* = 0.685), pharmacy (*p* = 0.455), physiotherapy (*p* = 0.308) and podiatry (*p* = 0.377). For psychology and social work, the range of frequencies across towns was so small (3.4% and 1.5%, respectively) that the GLMM algorithm dropped the random effect from the model. That is, town was redundant and uninformative in these two models.

Most of the models performed well, with only those for pharmacy and physiotherapy failing to classify correctly on more than 60% of occasions (Table 6). Specificity exceeded sensitivity for physiotherapy alone, indicating that these models were slightly better at predicting no use of the service when that is in fact the case than they were at predicting the use of the service when it was used. The opposite applies to the other modalities, although the balance is almost even for social work.

**TABLE 6:** Classification performance of each of the models.

	AUC [95% CI]	Accuracy (%)	Specificity (%)	Sensitivity (%)
Audiology	0.736 [0.710, 0.762]	62.58	60.74	73.53
Exerc. physiol.	0.741 [0.678, 0.804]	66.30	66.17	70.97
Optometry	0.722 [0.701, 0.742]	67.62	65.18	70.49
Pharmacy	0.625 [0.601, 0.649]	57.57	55.67	61.48
Physiotherapy	0.591 [0.561, 0.620]	57.92	58.62	54.76
Podiatry	0.769 [0.743, 0.795]	70.76	70.55	72.30
Psychology	0.735 [0.697, 0.772]	62.65	61.89	73.13
Social work	0.707 [0.643, 0.770]	67.22	67.19	67.86

Abbreviation: AUC, area under the receiver operating characteristic curve.

## 4 Discussion

Two allied health services, optometry and pharmacy advice, were used notably more frequently than the others, but GPs accounted for a far greater proportion of consultations than any allied health service did. The models of use of allied health services classified well. The sociodemographic variables associated with audiology and optometry were broadly similar, as were those for physiotherapy and exercise physiology. Psychology was particularly well used by those not working and those unable to work, and pharmacy advice was strongly sought by those unable to work. Podiatry use increased markedly with age. Trends were consistent across the four towns.

The typical user of audiology services was older, male and had private health insurance. An earlier study on the same geographic region as this, and Crossroads I (2000–2003), identified significantly greater odds of males with hearing difficulty, hearing problems identified by a doctor, tinnitus and requisite ear surgery or a procedure [9]. It can be postulated that such differences relate to occupational proclivities, especially farming and heavy industry. Hearing problems in a large survey of urban and rural Western Australia revealed that rural patients who presented for hearing screens had greater odds of hearing loss than those from urban areas, and men reported hearing loss more frequently than women from 50 to 69 years of age, corresponding roughly to the latter part of a working a working life [24]. Further, the rate of hearing loss in both sexes increased steadily with age, which accords with the observed use of audiology services in the present study, where the odds of using an audiologist increased 1.6‐ and 6.3‐fold for 10‐ and 40‐year increases in age, respectively.

As with audiology, optometry was characterised by an increase in use with age and greater use by women and those with private health insurance. Further, retired people had greater odds of using the service than those employed full‐time. The greater use by women has been observed as part of the World Health Organization's Global Burden of Disease study [25]. This found that globally females have greater incidence of mild vision impairment, moderate and severe vision impairment, and blindness in females, and the same trend was observed for the restricted case of Australasia, save moderate and severe impairment, for which no difference was observed [25]. There are manifold reasons behind these sex differences. Dry eye is a major eye health concern, and it affects women over the age of 50 at roughly twice the rate as men over 50, a difference in large part associated with menopause and other hormone‐related conditions, and women's lacrimal gland function is downregulated during pregnancy, after menopause, and during oral contraceptive use [26, 27]. Rheumatologic conditions are more common in women and can lead to an array of eye problems, such as corneal melt, retinitis, glaucoma, cataract, scleritis and uveitis [28]. Declining oestrogen with age leads to a greater risk of glaucomatus damage to the optic nerve; about 5%–8% of pregnant women with pre‐eclampsia suffer light sensitivity, lost/blurred vision, or visual auras; diabetic retinopathy increases in severity during pregnancy [28]. In addition to such physiological challenges, ocular trauma arising from domestic abuse is far more common in women [28, 29, 30, 31]. In most instances, retired people are likely older and in a reasonably sound financial situation, giving them both the increased need and means for using optometry services, and this might explain in part the greater odds of use observed here for this group.

A review of studies on access to health services in Australia for culturally and linguistically diverse Australians found that at the level of the individual and the family, major challenges to access included low health literacy and multimorbidity, *inter alia*. At the community level, acculturation was the primary factor, leading in turn to problems such as unhealthy lifestyles and communication barriers [32]. Here, home English speakers had greater odds of using physiotherapy than those who did not have English as their primary language at home. The reason for this is unclear. It may be related to people's familiarity with the service. Europe and the Americas have 1078 and 688 physiotherapists per million people, respectively, whereas Africa and Asia/Western Pacific have only 15 and 58, which equates to Africa hosting just 1% of the world's physiotherapists [33]. Physiotherapy would therefore likely be an unfamiliar health service to someone coming to Australia from Africa, and likewise for someone from Asia. The greater use of physiotherapy and exercise physiology by those unable to work relative to full‐time workers might reflect the use of manual‐, lifestyle‐ and exercise‐based interventions in chronic pain relief by those whose condition prevents them from working. In the Crossroads II study, 30.5% and 5.1% of persons in chronic pain used physiotherapy and exercise physiology compared with 14.2% and 2.2% for those not in chronic pain [34]. Similarly, psychology and social work saw not only greater odds of use by those permanently unable to work and by those who were neither working nor retired but also greater odds for those in chronic pain (psychology: 10.8% for those with chronic pain compared with 5.6% for without; sociology: 7.5% compared with 2.5%). Those unable to work also made a greater use of podiatry and advice from pharmacists, and these two services were also associated with more frequent use by those in chronic pain (podiatry: 17.6% for those with chronic pain compared with 11.7% for without; pharmacy: 43.8% compared with 29.6%). Health literacy may also play a role. Older people, females and those born in most countries outside Australia have been reported to have lower odds of having adequate health literacy [35].

Podiatry use is well known to increase with age [5, 6, 7, 36, 37]. Here, the use of podiatry increased markedly with age, with a yearly OR of 1.052 [1.039, 1.066], which equates to a 60‐year‐old having 7.6 times greater odds of using the service than a 20‐year‐old. The distribution was roughly the same as a national survey of the use of Medicare item 10 962 (podiatrist: mostly chronic conditions and complex care) from 2004 to 2008 [7]. Nationally, those over 65 years of age account for 75% of podiatry consultations, and here those over 65 accounted for 66% of those using podiatry. No sex difference was observed, though, which contrasts with the national survey, which found that females account for 63% of podiatry consultations [7].

Advice from a pharmacist is an important service, with one‐third of participants using it. Such an arrangement demands confident and efficient communication skills of both the pharmacist and the patient. This may in part explain the greater odds of use observed for those with a bachelor's degree and those born in Australia, although there was no difference between those who had English as their primary language at home and those who did not. Those unable to work also made a greater use of pharmacy advice, and this might be related to a financial imperative, chronic pain or both. Pharmacists have been well documented to provide counselling to women on pregnancy and breastfeeding, and this might contribute to the sex difference observed here [38, 39, 40, 41].

This study permitted assessment of associations and could not link cause and effect, which would require an experimental approach. Each response variable was the use of an allied health modality. A more complete understanding might emerge from questions relating to the quality of the services. There were no data available on the financial situation. These could be included in future studies. The study was restricted to towns. I fuller representation of the rural setting would include those on farms.

Allied health services are pivotal to the operation of an effective health system in Australia, yet little is known about the sociodemographic factors associated with the use of allied health in regional Australia. The analyses presented here demonstrate various clear associations for eight allied health services. Although the factors associated with use varied across modalities, a notable commonality was that those unable to work had greater odds of using six of the eight services investigated. This study offers an insight into the use of allied health services in a rural region of Australia, but regional Australia varies considerably geographically and sociologically, and more studies are required to paint a higher resolution picture across regional Australia.

## Author Contributions

**Andrew J. Hamilton:** formal analysis; data curation; writing – original draft. **Ryan McGrath:** methodology; formal analysis; writing – review and editing. **Lisa Bourke:** conceptualization; funding acquisition; writing – review and editing; formal analysis. **Kristen M. Glenister:** data curation; methodology. **David Simmons:** methodology; conceptualization; funding acquisition; writing – review and editing.

## Ethics Statement

Ethics approval was granted by the Goulburn Valley Health Ethics Committee (GVH 20/16) in May 2016.

## Consent

Informed written consent was obtained from each household survey participant aged 16 years or older.

## Conflicts of Interest

The authors declare no conflicts of interest.

## Data Availability

The data that support the findings of this study are available on request from the corresponding author. The data are not publicly available due to privacy or ethical restrictions.
